# Supplementary material for: Gaps, traps, bridges and props: a mixed-methods study of resilience in the medicines management system for patients with heart failure at hospital discharge
Source: BMJ Open. 2019 Feb 5;9(2):e023440. doi: 10.1136/bmjopen-2018-023440 (PMC6377507; doi:10.1136/bmjopen-2018-023440)
Supplement: Author's manuscript [file bmjopen-2018-023440.draft_revisions.pdf]

BMJ Open is committed to open peer review. As part of this commitment we make the peer review history of every article we publish publicly available.

When an article is published we post the peer reviewers' comments and the authors' responses online. We also post the versions of the paper that were used during peer review. These are the versions that the peer review comments apply to.

The versions of the paper that follow are the versions that were submitted during the peer review process. They are not the versions of record or the final published versions. They should not be cited or distributed as the published version of this manuscript.

BMJ Open is an open access journal and the full, final, typeset and author-corrected version of record of the manuscript is available on our site with no access controls, subscription charges or pay-per-view fees (http://bmjopen.bmj.com).

If you have any questions on BMJ Open's open peer review process please email info.bmjopen@bmj.com

BMJ Open

Gaps, traps, bridges and props: a mixed-methods study of resilience in the medicines management system for heart failure patients at hospital discharge

Journal:	*BMJ Open*
Manuscript ID	bmjopen-2018-023440
Article Type:	Research
Date Submitted by the Author:	06-Apr-2018
Complete List of Authors:	Fylan, Beth; University of Bradford, School of Pharmacy and Medical Sciences<br>Marques, Iuri; University of Bradford, School of Pharmacy and Medical Sciences<br>Ismail, Hanif; University of Bradford, School of Pharmacy and Medical Sciences<br>Breen, Liz; University of Bradford, School of Pharmacy and Medical Sciences<br>Gardner, Peter; University of Leeds Institute of Psychological Sciences<br>Armitage, Gerry; Bradford District Care NHS Foundation Trust; University of Bradford, Faculty of Health<br>Blenkinsopp, Alison ; University of Bradford, School of Pharmacy and Medical Sciences
Keywords:	Resilience, Patient Safety, Heart failure < CARDIOLOGY, Medicines Optimisation, Quality in health care < HEALTH SERVICES ADMINISTRATION & MANAGEMENT

SCHOLARONE™
Manuscripts

Gaps, traps, bridges and props: a mixed-methods study of resilience in the medicines management system for heart failure patients at hospital discharge

Beth Fylan,[1] Iuri Marques,[1] Hanif Ismail,[1] Liz Breen, [1] Peter Gardner,[2] Gerry Armitage,[3] Alison Blenkinsopp[1]

[1] University of Bradford, Bradford, UK

[2] University of Leeds, Leeds, UK

[3] Bradford District Care Trust, Bradford, UK

Corresponding author: Dr Beth Fylan, School of Pharmacy and Medical Sciences, Richmond M24, University of Bradford BD7 1DP

Email: B.FylanGwynn@bradford.ac.uk

Tel: 01274 236952

Fax: Not available

Keywords: Patient safety; resilience; cardiology; medicines management

Word count - excluding title page, abstract, strengths and limitations, references, figures, tables, acknowledgements: 4,404

Abstract

Introduction

Poor medicines management places patients at risk, particularly during care transitions. For patients with heart failure (HF), optimal medicines management is crucial to control symptoms and prevent hospital readmission. This study explored the concept of resilience using HF as an example condition to understand how the system compensates for known and unknown weaknesses.

Methods

We explored resilience using a mixed-methods approach in four healthcare economies in the north of England. Data from hospital site observations, healthcare staff and patient interviews, and documentary analysis were collected between June 2016 and March 2017. Data were synthesised and analysed using framework analysis.

Results

Interviews were conducted with 45 healthcare professionals, with 20 patients at three time-points and 189 hours of observation were undertaken. We identified four primary inter-

For peer review only

related themes concerning organisational resilience. These were named as gaps, traps, bridges and props. Gaps were discontinuities in processes that had the potential to result in poorly optimised medicines. Traps were features of the system that could produce errors or unintended adverse medication events. For example, the need to expedite discharges as quickly as possible appeared to impact on the effective preparation of discharge information. 'Bridges' were features of the medicines management system that promoted safety and continuity which ensured that, despite varying conditions, care could be delivered successfully. 'Props' were informal, temporary or impromptu actions taken by patients or healthcare staff to avoid potential adverse events.

Conclusion

The numerous opportunities for HF patient safety to be compromised and medicines managed sub-optimally during this common care transition are mitigated by system resilience. Cross-organisational bridges and temporary fixes or 'props' put in place by individuals, (including patients and carers), teams and organisations are critical to the opportunity for safe and optimal care to be delivered in the face of continued system pressures.

Strengths and limitations of the study

- Using four geographical areas and mixed methods this study explores medicines management for people with heart failure at a time of risk in their care.
- Multiple viewpoints of patients and staff highlighted how the system is resilient in the face of pressure and weaknesses that places patients at risk.
- The study presents a novel framework within which to explore and understand resilience in healthcare systems: that of 'bridges' and 'props' set against a backdrop of 'gaps' and 'traps'.
- The study collected multiple viewpoints but did not include the perspectives of local, regional and national policy makers.
- The patients and staff who agreed to be interviewed may have had particularly positive or negative experiences of the system, although their accounts were augmented and triangulated by first-hand independent observations.

For peer review only - http://bmjopen.bmj.com/site/about/guidelines.xhtml

Introduction

Patients are placed at risk through poor medicines management, particularly during care transitions, such as hospital discharge,[1] when the responsibility for their care managed across organisations and clinicians, systems and processes are not optimally calibrated to manage the transition.[2] The World Health Organisation (WHO) views the safe management of medicines as a global challenge,[3] and UK guidance stresses the importance of improving the way medicines are managed at care transitions.[4]

Medicines management is a system that supports the therapeutic use of medicines by patients, involving multiple healthcare organisations and staff with different clinical specialties and professional roles.[5] There is no shortage of evidence about the points at which healthcare systems fail to provide safe care.[6-9] Patients are not always well prepared to leave hospital and self-manage their ongoing treatment.[10] The effective transfer of sufficient and accurate information between healthcare organisations remains inadequate in many cases,[2] compounded by boundaries between care providers who may not always have access to the same information about patients' health. It is then unsurprising that discrepancies arise between medicines lists held by different care providers and patients.[1 11 12]

Heart failure (HF) is a chronic progressive condition affecting 900,000 individuals in the UK and is projected to rise significantly with an ageing population.[16] HF is the second most costly condition for the NHS after stroke and is characterised by high rates of readmissions.[17] Hence the optimal management of medicines when leaving hospital is crucial to enhance quality of life, manage symptoms and prevent deterioration and hospital readmission and reduce mortality.[18]

Current thinking in patient safety has shifted focus from the deconstruction of events leading up to errors (Safety I) to a more positive and proactive view of healthcare systems that identifies and values what goes right as well as pinpointing what goes wrong (Safety II).[19 20] Thus Safety II focuses on preventing error whilst accepting that there is variability in the delivery of healthcare, acknowledging that patients do not routinely experience harm as a consequence of their care. It offers recognition of good performance in the face of uncertainty, valuing flexibility, adaptability, foresight and knowledge of how systems operate.[19 21] This in turn promotes a more dynamic attitude to performance through resilience which we define here as the ability for a system and the individuals therein to bounce back after any disruption or failure or in the face of ongoing, sustained pressure.[22]

For peer review only - http://bmjopen.bmj.com/site/about/guidelines.xhtml

This concept of resilience in healthcare has looked at specific risks such as handover of care between staff in one location such as a ward or performing specific roles, [21 23 24] and not on a point of transition between healthcare organisations, although one study explored how patients can enhance resilience in medicines management at and after hospital discharge through anticipating discrepancies and taking remedial action.[25] No studies to date have explored resilience in medicines management at this care transition from multiple perspectives, including staff and patients across different healthcare economies.

This study aims to address this evidence gap by systematically investigating resilience in the medicines management system, using HF as an example condition. More specifically, the study was designed to understand how the system compensates for weaknesses in order to deliver safe yet optimal treatment. Its objectives were to explore the system of medicines management in multiple healthcare economies to highlight where resilience in the exists and to identify where improvements to the system can be made to enhance resilience.

Methods

We used a mixed-methods design in four healthcare economies in the north of England. Data from site observations, staff and patient qualitative interviews, and documentary analysis (discharge letters and organisational and national policies) were collected between June 2016 and March 2017. NHS research ethics committee approval was sought and granted (16/NS/0018).

Patient involvement

A patient researcher was a member of the research team advising on patient recruitment, data collection materials and information and consent forms. The research was overseen by a patient-led steering group including people with heart failure and carers.

Data Collection

Observations

Following ward-level consent, three experienced health researchers (BF, HI, IM) conducted a total of 189 hours of observations in five cardiology wards and one heart failure clinic. Structured observation schedules developed by the research team informed by previous work[26] were used to record observations. We observed medicines and ward rounds, preparation of information for discharge, patient discharges, as well as any other impromptu medicines-related activities. Unstructured, contemporaneous field notes were taken by the researchers.

For peer review only - http://bmjopen.bmj.com/site/about/guidelines.xhtml

Patient recruitment

A quota sample of between 16-24 admitted patients was constructed to allow for attrition, aiming for 16 complete datasets. Patients were recruited during hospital admission by research nurses in consultation with ward staff. Patients were eligible for the study if they were aged 18 years or older, had capacity to consent, and had been admitted to hospital with a diagnosis of heart failure with reduced ejection fraction (<45%) measured by an echocardiogram within the last five years. In order to be eligible to participate, patients also needed to present New York Heart Association (NYHA) Class III symptoms.[27] Research nurses approached eligible patients to introduce the study. Patients were then provided with a participant information leaflet and given the opportunity to ask questions about the study; they were given at least four hours to decide whether or not to take part.

Patient interviews

Interviews at three timepoints explored patient experiences with their medicines from hospital admission to discharge at, or as soon as practicable, after discharge and again approximately two and six weeks later. The research team developed a semi-structured interview schedule built upon previous work[26] and a review of relevant literature. The schedule comprised questions relating to patients' experiences with their medicines, and prompts and probes were used when relevant. Two researchers conducted the interviews (BF, HI). Interviews lasted up to 60 minutes, took place in patients' homes and were video or audio recorded and transcribed verbatim.

Healthcare staff recruitment

Healthcare professionals with a role in medicines management in primary or secondary care were approached to take part in a semi-structured interview either by research nurses or the study team, using face-to-face communication or by e-mail invitation.

Healthcare staff interviews

An interview schedule was developed by the research team, to explore staff perceptions of the safety of medicines management. The schedule focused on medicines management processes, staff views on its quality and effectiveness for patients with heart failure in primary and secondary care, and their experiences of medicines management at discharge from secondary to primary care. Staff were given a participant information leaflet describing the study and, if they agreed to take part, an appointment was made to conduct the interview. Interviews lasted up to 60 minutes, were audio-recorded following written consent and transcribed verbatim.

For peer review only - http://bmjopen.bmj.com/site/about/guidelines.xhtml

Analysis of key documents

Documents were identified and reviewed including: national guidance on medicines optimisation used in the hospital setting;[4] local policies on medicines management and discharge in the four health economies; and any patient information about medicines in use in the four hospitals and available as text. Examples of system resilience at care transitions and risks in the system were extracted using a framework that mapped them according to the point in the transition to which they related and to the resilience element (or lack of) they evidenced.[21]

Data analysis

The process of data analysis was iterative and comparative: analysing the first round of interview and observation data as further interviews and observations were undertaken; providing the opportunity to explore emerging themes in greater detail in subsequent fieldwork. The research team met several times to discuss the data synthesis and analysis method and the emerging themes. Interview data were synthesised through data extraction with the data from observations and documents and the combined data were analysed using the Framework approach,[28] involving detailed familiarisation with the data, identifying themes, interpreting the findings within the context of similar research studies, and considering policy and practice. The emerging analysis was thematic.

Results

A total of 56 interviews with 20 heart failure patients were conducted: nineteen at discharge or shortly afterwards (timepoint 1); 19 approximately two weeks after discharge (timepoint 2); and 18 approximately six weeks after discharge (timepoint 3). We were unable to contact one patient from site 1 at time-point 1; at site 2 one patient withdrew from the study after the first interview. One patient from Site 3 was not interviewed at the third time-point due to hospital readmission, and at site 4 one patient was too ill to continue after the second interview. Table 1 presents the number of patients interviewed for each site at the different time points. Table 2 outlines the gender and age of interviewed patients.

Table 1: The number of patients interviewed at each time-point by site

Site	Timepoint 1	Timepoint 2	Timepoint 3
**Site 1**	2	3	3
**Site 2**	5	4	4
**Site 3**	6	6	5
**Site 4**	6	6	5

For peer review only - http://bmjopen.bmj.com/site/about/guidelines.xhtml

Total	19	19	18

Table 2: The gender and age of patients who took part in interviews

Site	Gender	Total	Age range
**Site 1**	Male	2	72-82
	Female	1	53
**Site 2**	Male	5	40-89
	Female	0	0
**Site 3**	Male	5	46-79
	Female	1	69
**Site 4**	Male	4	46-78
	Female	2	69-76

Forty-five interviews, detailed in Table 3 were conducted with healthcare professionals: 19 with primary care staff in four GP surgeries and 26 with secondary care staff. Table 4 presents the number of healthcare staff interviewed by site.

Table 3: Number of interviews by healthcare staff type

Staff Type	Number of interviews
GPs	4
Practice administrators / data quality managers	2
Practice pharmacists	3
Community pharmacists	2
Practice nurses	1
Community heart failure nurses	2
Practice managers	3
Clinical care co-ordinators	1
Community cardiac nurses	1
Cardiologists	3
Ward managers	5
Staff nurses	2
Junior sisters	1
Ward pharmacists	3
Specialist cardiology pharmacists	2
Consultant pharmacists	1
Junior doctors	2
Specialist heart failure nurses	3
Ward administrative staff	4
Total	**45**

For peer review only - http://bmjopen.bmj.com/site/about/guidelines.xhtml

Table 4: The number of healthcare staff interviewed per site

Site	Primary/community care	Secondary care
**Site 1**	6	7
**Site 2**	4	8
**Site 3**	2	4
**Site 4**	7	7
**Total**	**19**	**26**

Results

We identified four primary inter-related themes concerning organisational resilience: These were: gaps, traps, bridges and props. Examples representing each theme are shown in Tables 5-8.

'**Gaps**' were defined as a discontinuity in key processes that form the medicines management system and had the potential to result in poorly optimised medicines. Approaches to preparing discharge information varied across sites, with information sometimes being missed due to a lack of preparation time. Gaps were also evident in the information shared with primary care and in the preparation of patients to use their medicines. For the latter, we identified no standardised processes for informing patients about their medicines and, while hospital policies stipulated that patients should be informed, and gave details of the types of information patients should have, there was no guidance on optimal methods for informing patients about their medicines or training in doing so.

> *Discussions with some nurses during observations revealed that while they were aware of policies in place on what aspects to cover when discussing medicines with patients at discharge, they did not follow them and often rushed these conversations [Site 2 - Field notes from ward observations]*

> *Yes, you learn [how to give information about discharge medicines] from someone else. There's no course that you go on to say, 'This is what we do and why'. You can understand the rationale for being more thorough and why we do the things we do, but in terms of formal training, we don't have any* [Ward Nurse, Site 1].

After discharge we found gaps in the continuity of care, for example not all patients had a community pharmacy Medicines Use Review (MUR) because pharmacies did not routinely receive information about the patients' medicines at discharge. Waiting times for specialist follow-up varied considerably and were sometimes lengthy, for example waiting times for an

For peer review only - http://bmjopen.bmj.com/site/about/guidelines.xhtml

appointment with community heart failure specialist nurses who would manage medicines titration was sometimes as long as three months after discharge.

We defined '**traps'** as features of the way the medicines management system was designed or managed that might produce errors or unintended adverse medication events. These were evident in the co-ordination of discharges, for example the pressure on ward staff to expedite discharges as quickly as possible appeared to impact on the effective preparation of discharge information and on educating patients about their discharge medicines.

> *If you're busy, you'll write less and I think that's just what happens on the job. If I know I've got time, I'll make sure I input as much detail as possible, but if you're busy you just don't have the time to do that, so you'll just really do short summaries and just include the bare essentials. (Site 1 FY1 doctor)*

Staff preparing discharge information were interrupted, could not always locate patients' notes and none reported receiving training about safe practices with medicines at discharge to primary care. We also found error traps after discharge, such as a lack of time and resources in GP surgeries to process discharge information. Finally, there was evidence that patients' lack of knowledge about the purpose of their medicines could potentially cause confusion particularly when the changes made in hospital led patients to have different supplies or multiple multi-compartment 'compliance aid' tablet boxes. Like hospital staff, none of the primary care staff had received formal training about safe practices with medicines at discharge to primary care.

'**Bridges'** were identified as formal features of the medicines management system that promoted the safety and continuity of medicines management. They ensured that, despite varying conditions, care could be delivered successfully to heart failure patients.

> *When preparing the "To Take Home" medicines at discharge, ward staff wait for the pharmacist to come to the ward to check the patients' medicines lists and ensure these are accurate and any errors can be rectified.* [Site 2 - Field notes from ward observations]

Bridges also included methods of communicating with primary care about treatment, for example, when hospitals sent an electronic copy of the patient's discharge summary to their general practitioner (GP). In this case, summaries were put together by multidisciplinary teams including junior doctors, nurses and pharmacists who would check and add information about medicines that would be useful to the primary care team. After discharge,

For peer review only - http://bmjopen.bmj.com/site/about/guidelines.xhtml

two participating hospital trusts ran pharmacist-led titration clinics to ensure that medicine doses were optimised. Titration clinics also meant patients would be seen more quickly than if they had to see a consultant. One cardiology pharmacist explained that the titration clinic ensured patients' medicines were adjusted as and when appropriate, in light of some GPs not feeling confident about changing them: .

GP practices differed in how they processed discharge information. In one practice, administrative staff would review the discharge summary and forward actions to practice staff if medicines information needed to be changed. In another practice, this task was the responsibility of the GP, who would forward actions to practice staff and book any tests as a consequence of any changes in medicines occurring during hospital stay (i.e. blood tests). One GP reported that his practice had re-engineered their processing of discharge summaries to include a multidisciplinary team comprising administrative staff, a practice-based pharmacist (whose post was created in response to recognised safety risks) and GPs, with the pharmacist taking responsibility for coordinating the process.

> *"And so [processing discharge summaries] it was in-between surgeries, it was at the end of the day, so it was being fitted in rather than having allocated time, so naturally when it's being fitted in the process is a bit more rushed, you're more under pressure, maybe your concentration levels aren't there, so mistakes can be easily made. So as a practice we made the decision that just in terms of a workload thing and also patient safety and efficiency it would be worth investing in sort of pharmacy services."* [GP, Site 1]

Practice pharmacists also reported perceiving that their specialist knowledge improved as a consequence of being involved in the discharge process, while further expediting the safe management of medicines for patients after discharge. Some practice staff described having targets in place linked to time taken to process discharge summaries, with some practices prioritising processing driven by the risk of readmission. One data quality manager explained that they tried to process discharges within 24 hours, including reconciling medicines, but also explained that they had a maximum of a week to complete it.

> *"So we have a week turnaround in order to get any meds reconciliation done. We generally get our electronic discharge normally within 24 hours of the patient being discharged, that would be scanned through the system that will then go to the doctor, the doctor will then forward it to me generally for coding and also to our practice pharmacist."* [Data quality manager, Site 2]

'**Props'** were informal**,** temporary or impromptu actions taken by patients or healthcare staff to avoid potential adverse events, such as medication errors. Props were sometimes

For peer review only - http://bmjopen.bmj.com/site/about/guidelines.xhtml

developed in response to risks in the working environment, such as interruptions during medicines rounds.

> *During medicines rounds, nurses are frequently interrupted whilst sorting patients' medicines. One nurse observed also uses the strategy of signing the drug chart soon as one medicine is sorted into the plastic cup before moving to the next medicine. If there are interruptions, the nurse will know which medicines have already been sorted by looking at the drug chart. The nurse says it is a brilliant strategy to ensure accuracy and safety and cope with inevitable distractions and interruptions* [Site 1, Field notes from ward observations]

Hospital staff told us that they suspected recommendations made by the hospital (for example the up-titration of doses which is critical in HF) may not be acted on in primary care, due to loss of information or lack of expertise. Hence, staff created solutions to prevent a break in the ongoing treatment, giving patients an extra copy of their discharge letter to take to the GP. Some staff members described being cognisant of how discharge information can be difficult for patients to understand and would take extra time to explain the discharge summary and any abbreviations contained within it. One staff nurse at Site 1 described having to make protected time to hold these discussions with patients, drawing curtains around the patients' beds to prevent any disruption. Some patients reported being discharged with an insufficient amount of medicines, leading them to seek community pharmacists help to provide them with emergency supplies until they could see a GP. Some patients also proactively provided the necessary links between community pharmacy, general practitioners and the hospital after discharge. For example, one patient called the community pharmacy to ask what information, if any, they had been provided with about his medicines. Another patient provided their GP practice with information about dose changes.

> *So when I'd run out, I rang my GP and they were blissfully unaware of any changes to the amount, the receptionist had to take it down. She says "well what was you on?" and I said "well I was on one tablet a day and then they took me down to half, then they put me to one tablet a day again and now I'm on two tablets a day" "Two tablets?", this is the receptionist's questions. I says "yeah, two tablets." (Patient 05, Site 4, interview 2)*

Finally, community pharmacists stepped in to organise supplies for patients when something had gone wrong and the patient was unable to get the correct medicines.

For peer review only - http://bmjopen.bmj.com/site/about/guidelines.xhtml

Table 5: Gaps at and after hospital discharge

	At discharge	After discharge
**Gaps**	Discussions about medicines at discharge can be rushed, due to time pressures and workload.	Community pharmacy is not integrated into communication about discharge medicines
	No standard process or guidance on how to hold discussions with patients about medicines	Patients are not routinely referred to community pharmacy for follow-up support
	Limited or no formal training about care transitions, preparing discharge summaries or patients to use medicines for all staff	Limitations to the extent of shared IT systems between primary and secondary care and between surgeries and pharmacies
	Processes for preparing patients to go home with medicines are linear but not streamlined, for example multiple staff members need to input which causes delays	Not all surgeries have a practice pharmacist~~s~~ to reconcile medicines
	Discharge summary information is technical and uses jargon and abbreviations which are difficult for patients to understand	Long waiting times to access community heart failure nurse services (up to 12 weeks)
	Inconsistency in level of detail in information written on discharge summary due to workload and HCP knowledge of the patient	Some patients perceive limitations in post-hospital follow-up care, including difficulty in accessing services in primary care
	Varying information offered to patients about follow-up appointments	Patients are not fully aware of the roles and skills of primary care staff, particularly community pharmacists
	Limited awareness among staff about policies in place for medicines management	Some patients unable to devise effective strategies to self-manage medicines at home
	Effectiveness of discharge are not critically appraised due to lack of feedback (unless the patient is readmitted or primary care staff make queries)	

Table 6: Traps at and after hospital discharge

	At Discharge	After discharge
**Traps**	Patient knowledge of medicines when they are discharged is limited	Community pharmacy does not routinely receive copies of patients' discharge summaries so cannot correct or query new GP prescriptions
	There is pressure on ward staff to discharge patients and free-up beds	Patients have an on-going lack of knowledge of their medicines once home
	Variation in ward staffing levels and varying numbers of discharges to perform each day	No formal training for surgery staff to process discharge information
	Use of several different IT systems in producing information for discharge	Lack of time and resources in surgery to process discharge information
	Staff preparing patients for discharge and information about discharge medicines are interrupted	Systems allow old prescriptions to be issued when medicines have changed
	Preparing information for discharge routinely left to junior members of staff who may not be familiar with the patient	Dosages are monitored and changed by HCPs in different organisations
	Conversations about medicines with patients at discharge can be left to the last minute	Trust in healthcare professionals may lead to a lack of critical appraisal of one's condition and medicines
	Patients transferred to discharge lounges to await medicines face an extra transfer of care	Changes in medicines lead to patients having conflicting medicines and MCCA boxes at home
		Varying levels of communication across care organisations results in extra burden to patient who has to fill in the gaps
		Varying information about medicines changes provided to primary care may lead to HCPs having to make

For peer review only
For peer review only - http://bmjopen.bmj.com/site/about/guidelines.xhtml

		decisions based on assumptions
		Healthcare professions may not accept treatment recommendation by other healthcare professionals (e.g. GP not accepting recommendations made by HFSN)

Table 7: Bridges at and after discharge

	At discharge	After discharge
**Bridges**	Hospitals have established methods of communicating about patients' treatment with primary care	Some trusts provide outpatient clinics where patients can receive IV fluids, thus avoiding them to need to be admitted to receive these medicines or speeding up discharges
	Preparing discharge summaries and TTO lists is a multidisciplinary task involving nurses and pharmacists	GP practices have systems for acting upon discharge information once it is received, although processes and times to process this information vary
	Ward pharmacists can expedite well managed discharge through proactively creating TTO lists	Some practices have targets in place linked to time to process discharge information (e.g. 24h from receiving this information)
	One trust routinely referred patients to community pharmacy for follow-up support with their medicines	One practice pharmacist reengineered the process for action on discharge information
	All hospitals had policies for informing patients about their medicines	Some practices use practice pharmacists to improve an expedite the processing of discharge information
	Heart failure nursing staff attempted to see patients before their discharge to talk about their medicines to avoid having these conversations rushed at discharge	Community pharmacy is sometimes able to perform post-discharge Medicines Use Reviews for Patients
	In two trusts, ward-based pharmacists would speak to patients about their medicines before discharge	Two hospital trusts run pharmacist-led titration clinics to manage patients' medicines, meaning that patients can be seen and followed-up quickly
	Patients received written information about their medicines, with one trust providing an easy-to-understand medicines chart occasionally annotated by staff	Some practices have ambulatory services
	Patients are referred to specialist heart failure teams for follow-up	Heart failure specialist nurses offer support services including medicines optimisation
		Some GP practices have systems to identify discharged patients with high risk of being readmitted so they can take preventative action

For peer review only

For peer review only - http://bmjopen.bmj.com/site/about/guidelines.xhtml

Table 8: Props at and after hospital discharge

	At discharge	After discharge
**Props**	Some Staff create their own checklists to follow discharge processes, such as using the discharge summary to tick off medicines	Patients create their own lists of medicines, going online to seek more information
	Staff occasionally give patients two copies of the discharge summary so that patients can give one to their GPs in case they do not receive it electronically	Community pharmacists who have received a copy of the discharge summary use them to check against repeat prescriptions before dispensing
	Staff make ad-hoc queries to establish reasons for medicines changes which are unclear and undocumented so that they can be clear on the discharge summary	Patients check medicines prescribed by their GPs against their discharge summary and/or take a copy when go see the GP or update them verbally
	Staff will delay discharge to wait for relatives to arrive so that they can include them in conversations about medicines	GP identifying potentially problematic changes in medicines occurring in hospital due to their enhanced knowledge of the patient
	Ward pharmacists give advice to patients if they are concerned about patients getting confused, for example, advising them to return their old medicines to the pharmacy for disposal and only take the new ones	GPs try to fill in patients' knowledge gaps about their medicines after discharge
	Patients write additional information on the medicines' boxes or ask staff to write it so that they can better manage their medicines at home, for example time to take medicines	Community pharmacy provides emergency supply of medicines when patients are discharged from hospital without sufficient medicines
	Patients are sometimes cognisant of how difficult it is for patients to understand their medicines and information provided at discharge, so they take extra time to hold these conversations	Patients are given telephone numbers for heart failure nurses to contact them after discharge because waiting times to be seen by them are long
	Staff draw curtains around the patients' beds when talking to them to ensure privacy and prevent interruptions	Heart failure nurses can identify where patients make mistakes taking their medicines, for example, continuing to take discontinued medicines
	Nurses resist instructions to send patients to discharge lounges as they feel the staff will not have specialist knowledge, and provide enhanced instructions to discharge lounge if overruled	Heart failure nurses use the patients as a conduit for information to be exchanged between them and other healthcare professionals
	Junior doctors query with pharmacist on ward if they need additional information about medicines	Patients develop individual strategies and routines to adhere to medicines at home, for example alarms, writing additional information in the discharge summary, storage systems, affixing discharge summaries on the fridge, etc
		Some patients take all their medicines to community pharmacy after discharge, seeking information on which medicines they should continue to take and which should be discarded

For peer review only - http://bmjopen.bmj.com/site/about/guidelines.xhtml

Discussion

Notwithstanding a very considerable body of research that has illuminated the sometimes alarming level of preventable harm in healthcare systems and how this could be reduced,[29 30] this study demonstrates that patients still face safety threats through inadequate medicines management. The study also provides a positive perspective on the strategies developed and actions taken by healthcare organisations, healthcare staff, and patients to mitigate risk in the face of continued pressure. The multiple perspectives of patients and multidisciplinary healthcare staff, independent observation of practice and documentary analysis collected through mixed-methods allowed the possibility of triangulating data from multiple sources to offer a thorough description of a complex system. In doing so we also present a novel framework within which to explore and understand resilience in healthcare systems: that of ‘bridges’ and ‘props’ set against a backdrop of ‘gaps’ and ‘traps’. Moreover, this study explores a whole healthcare system in multiple settings inclusive of its transitions, revealing the context of that system. In contrast, previous studies although adding to an understanding of system resilience, have examined these problems from either a health professional perspective[23] or a patient perspective.[25]

Bridges and props either provided permanent solutions to potential gaps in care, or temporary fixes, usually implemented by individuals or small teams. Sometimes the props, were put in place to minimise the risk of error despite organisational pressure, for example to discharge patients and free beds. This study also draws out the dissonance between what healthcare professionals believe should happen and the reality of contemporary practice. This was clear from the differences between the recommendations for hospital discharge from national guidance and local policy, where, for example, patients must be fully informed about their medicines and any changes, and the overall discharge process – which in the settings observed, may lack depth or appear rushed or lacking the necessary detail. This was sometimes due to different local conditions, such as the number of discharges that needed to be completed in a day, but also to local policies that lacked sufficient detail and were not supported by staff training.

Resilient systems are able to anticipate threats, respond when errors or adverse events occur and learn from failures.[32] It was evident that staff were able to anticipate system vulnerabilities, for example in the transfer of discharge information, and take compensatory action in the form of ‘props’. As found by a previous study, patients also took remedial action, such as providing missing information about medicines changes to staff.[25]

Implications for policy and practice

For peer review only - http://bmjopen.bmj.com/site/about/guidelines.xhtml

Successive UK government-commissioned reports have highlighted how care systems have failed and how the actions – or inactions of those who lead or contribute to the system – have sometimes led to poor care and patient harm.[33-35] Policymakers should recognise and attempt to learn from the actions taken by individuals and healthcare teams to deliver safer care for patients despite a disconnected communication system, varying staffing levels and the under-provision of formal training, for example in discharge and care transfers. Our study has shown that improvements to both efficiency and safety of care could be improved through connecting the discrete IT systems that operate within and between organisations. Additionally, community pharmacists often remain isolated from the patient pathway and are not routinely included in the communication between secondary care and primary care practice, creating additional risk for heart failure patients who must obtain new supplies of critical medicines often within one or two weeks of being discharged.[2] Implementing systems that enable community pharmacists to know about medicines changes made during hospital admissions and thus to reconcile subsequent GP prescriptions would improve safety of medicines management, especially for heart failure patients whose medicines are very commonly changed following a period of acute care. Local electronic systems do exist in some areas to ensure that the dispensers of post-discharge medicines are fully informed about the medicines hospital clinicians intended patients should take so that they can reconcile those medicines and ensure accurate ongoing supplies.[37] Policy makers also have a duty to help disseminate and promote implementation of these local innovations – such as the transfer of discharge medicines information to all agents in the medicines management system – which minimise inherent risk.

Patients, if they so desire, should also be provided with the opportunity to gain in-depth knowledge of their medicines before leaving hospital (or afterwards if they prefer), in order to enhance their ability to self-manage and monitor their condition; such knowledge might also increase patients' vigilance, detecting errors and allowing earlier intervention if medication problems arise. Materials to support patients should be developed using co-design methods to maximise their acceptability and usability with both patients and healthcare staff.[38] Policy makers may also consider allowing patients to write to and share a personal health record to keep track of and flag problems they may have with their medicines, and share those issues with members of their healthcare teams and report them to regulators.[39] This would in some measure help address the underreporting of medication errors, particularly in primary care.[40]

We found that staff received little formal training in co-ordinating medicines management, including in completing discharge summaries, and there was little evidence of inter-professional or cross pathway training. This type of training for healthcare professionals

For peer review only - http://bmjopen.bmj.com/site/about/guidelines.xhtml

about care transitions may foster a care environment where clinical and administrative staff have a better appreciation of the impact of the care they provide on different parts of the system, and on different colleagues. For example, how inadequate information on a discharge summary can cause difficulties for primary care staff attempting to reconcile medicines. Additionally, in primary care, understanding that the processing of discharge information can impact on patients and community pharmacists who must take action to ensure the correct medicines are supplied. Inter-professional education has been found to yield positive outcomes in healthcare, although more evidence to its effectiveness has been called for.[41]

Implications for future research

The Safety I paradigm has produced valuable ways of unearthing and visualising risks within systems and explaining causation when accidents occur, for example Reason's Swiss Cheese Model, highlighting how latent conditions can lie dormant in systems until holes in defenses align to allow a hazard to become a loss.[32] Rasmussen's risk management framework (ACCIMAP),[42 43] has offered a way of graphically representing causal flow of events along with internal and external management and regulatory factors. A map of a resilient healthcare system across a care transition would allow a graphical view of how a healthcare system delivers safe care to patients in the face of disruption and pressure across its many levels – individual, micro and macro – to allow staff to understand their position in safe care, and commissioners to view how decisions about changes to services may impact on a complex system. We propose this as a ResiMap.

Limitations

We observed practice in four NHS Trusts and interviewed a wide-range of healthcare staff across the pathway and patients, alongside reviewing key documents, we did not include the perspectives of local, regional and national policy makers, which may have enhanced the understanding of how systems are designed and the gaps between design and delivery. Nevertheless we were able to collect a large amount of data to compare policy practice which enhanced reliability and validity. The patients and staff who agreed to be interviewed may have had particularly positive or negative experiences of the system, although their accounts were triangulated by first-hand independent observations. Finally, the study was conducted in four NHS healthcare economies, at a time of heightened focus on the quality of healthcare, and reports of unprecedented financial constraints, which may have impacted on people's perspectives of care received and delivered, and on the nature of the care observed.

For peer review only - http://bmjopen.bmj.com/site/about/guidelines.xhtml

Conclusion

There are numerous opportunities for patient safety to be compromised and medicines to be sub-optimally managed during this care transition. However, there are also cross-organisational bridges and temporary fixes in the form of props, put in place by individuals, including patients an carers, and teams to maximise the opportunity for safe and optimal care to be delivered. For example, some GP surgeries have systems in place to ensure the timely and efficient processing of discharge information. Investigating gaps and traps in the healthcare system and identifying existing compensatory props and bridges allow the illustration of areas where healthcare can be improved and fragmented communication minimised during care transitions.

Acknowledgements

We would like to thank the patients and NHS staff who took part in this research. We also with to thank other members of the ISCOMAT programme management team.

Funding statement

This study was funded by the National Institute of Health Research for funding this research programme Improving the safety and continuity of medicines management at care transitions (ISCOMAT) RP-PG-0514-20009. The views expressed are those of the authors and not necessarily those of the NHS, the NIHR or the Department of Health and Social Care.

Data sharing statement

The transcripts of interviews are not available at the Dryad repository

Contributors

BF drafted the protocol, developed data collection tools, conducted fieldwork, analysed the data and drafted the manuscript. IM conducted fieldwork, analysed the data and drafted the manuscript. HI conducted fieldwork, analysed the data and commented on the manuscript. LB advised on data analysis. PG drafted the protocol, directed the study, advised on data analysis and commented on the manuscript. GA designed the study, drafted the protocol, directed data analysis and drafted the manuscript. AB designed the study, drafted the protocol and edited the manuscript.

Competing interests

None declared.

For peer review only - http://bmjopen.bmj.com/site/about/guidelines.xhtml

References

1. Coleman E, Smith JD, Raha D, et al. Posthospital Medication Discrepancies. Prevalence and Contributing Factors. *Archives of internal medicine* 2005;165:1842-47.
2. Urban R, Paloumpi E, Rana N, et al. Communicating medication changes to community pharmacy post-discharge: the good, the bad, and the improvements. *International Journal of Clinical Pharmacy* 2013;35(5):813-20. doi: 10.1007/s11096-013-9813-9
3. The World Health Organization. Medication Without Harm: WHO Global Patient Safety Challenge: The World Health Organization, 2017.
4. National Institute for Health and Care Excellence. Medicines optimisation: the safe and effective use of medicines to enable the best possible outcomes (NICE guideline 5), 2015.
5. Phipps DL, Morris RL, Blakeman T, et al. What is involved in medicines management across care boundaries? A qualitative study of healthcare practitioners' experiences in the case of acute kidney injury. *BMJ Open* 2017;7(1):e011765. doi: 10.1136/bmjopen-2016-011765
6. Leape LL, Brennan TA, Laird N, et al. The nature of adverse events in hospitalized patients. Results of the Harvard Medical Practice Study II. *The New England journal of medicine* 1991;324(6):377-84. doi: 10.1056/NEJM199102073240605
7. Brennan TA, Leape LL, Laird NANM, et al. Special Articles Incidence of Adverse Events and Negligence in Hospitalized Patients Results of The Harvard Medical Practice Study I. *The new England Journal of Medicine* 1991(Appendix I):370-76.
8. Landrigan CP, Parry GJ, Bones CB, et al. Temporal trends in rates of patient harm resulting from medical care. *The New England Journal of Medicine* 2010;363(22):2124-34. doi: 10.1056/NEJMsa1004404
9. Waring J, Bishop S, Marshall F. A qualitative study of professional and carer perceptions of the threats to safe hospital discharge for stroke and hip fracture patients in the English National Health Service. *BMC Health Serv Res* 2016;16:297. doi: 10.1186/s12913-016-1568-2
10. Knight D, Thompson D, Mathie E, et al. 'Seamless care? Just a list would have helped!' Older people and their carer's experiences of support with medication on discharge home from hospital. *Health Expectations* 2013;16(3):277-91. doi: 10.1111/j.1369-7625.2011.00714.x
11. Duggan C, Feldman R, Hough J, et al. Reducing Adverse Prescribing Discrepancies Following Hospital Discharge. *International Journal of Pharmacy Practice* 1998;6(2):77-82. doi: 10.1111/j.2042-7174.1998.tb00920.x
12. Morcos S. Where are the weakest links?: A descriptive study of discrepancies in prescribing between primary and secondary sectors of mental health service provision. *Psychiatric Bulletin* 2002;26(10):371-74. doi: 10.1192/pb.26.10.371
13. Garfield S, Barber N, Walley P, et al. Quality of medication use in primary care--mapping the problem, working to a solution: a systematic review of the literature. *BMC medicine* 2009;7:50-50. doi: 10.1186/1741-7015-7-50
14. Blenkinsopp A. Evaluation of the Discharge Medicines Review Service - Chapter 1 Literature Review, 2014.
15. Forster AJ, Murff HJ, Peterson JF, et al. Adverse drug events occurring following hospital discharge. *Journal of General Internal Medicine* 2005;20(4):317-23. doi: 10.1111/j.1525-1497.2005.30390.x
16. Taylor CJ, Ryan R, Nichols L, et al. Survival following a diagnosis of heart failure in primary care. *Fam Pract* 2017;34(2):161-68. doi: 10.1093/fampra/cmw145
17. Stewart S, Jenkins A, Buchan S, et al. The current cost of heart failure to the National Health Service in the UK. *European Journal of Heart Failure* 2002;4:361-71. doi: 10.1016/S1388-9842(01)00198-2
18. Koshman SL, Charrois TL, Simpson SH, et al. Pharmacist Care of Patients With Heart Failure. *Archives of Internal Medicine*;168(7):687-94.
19. Braithwaite J, Wears RL, Hollnagel E. Resilient health care: turning patient safety on its head. *Int J Qual Health Care* 2015;27(5):418-20. doi: 10.1093/intqhc/mzv063
20. Hollnagel E. Making health care resilient: From Safety-I to Safety II. In: Hollnagel E, Braithwaite J, Wears RL, eds. Farnham: Ashgate Publishing Limited 2013.
21. Jeffcott SA, Ibrahim JE, Cameron PA. Resilience in healthcare and clinical handover. *Quality & safety in health care* 2009;18(4):256-60. doi: 10.1136/qshc.2008.030163

For peer review only - http://bmjopen.bmj.com/site/about/guidelines.xhtml

22. Barasa EW, Cloete K, Gilson L. From bouncing back, to nurturing emergence: reframing the concept of resilience in health systems strengthening. *Health Policy Plan* 2017;32(suppl_3):iii91-iii94. doi: 10.1093/heapol/czx118
23. Larcos G, Prgomet M, Georgiou A, et al. A work observation study of nuclear medicine technologists: interruptions, resilience and implications for patient safety. *BMJ Quality & Safety* 2017;26(6):466.
24. Furniss D, Barber N, Lyons I, et al. Unintentional non-adherence: can a spoon full of resilience help the medicine go down? *BMJ quality & safety* 2014;23(2):95-8. doi: 10.1136/bmjqs-2013-002276
25. Fylan B, Armitage G, Naylor D, et al. A qualitative study of patient involvement in medicines management after hospital discharge: an under-recognised source of systems resilience. *BMJ Qual Saf* 2017
26. Fylan Gwynn E. Medicines management after hospital discharge: patients' personal and professional networks. University of Bradford, 2015.
27. Zile MR. New Concepts in Diastolic Dysfunction and Diastolic Heart Failure: Part I: Diagnosis, Prognosis, and Measurements of Diastolic Function. *Circulation* 2002;105(11):1387-93. doi: 10.1161/hc1102.105289
28. Ritchie J, Lewis J. Qualitative research practice: a guide for social science students and researchers. London: Sage 2003.
29. Classen DC, Pestonik MS, Evans RS, et al. Adverse Drug Events in Hospitalized Patients Excess Length of Stay, Extra Costs, and Attributable Mortality. *JAMA* 1997;277(4):301-06.
30. Runciman WB, Roughead EE, Semple SJ, et al. Adverse drug events and medication errors in Australia. *International Journal for Quality in Health Care* 2003;15(Supplement 1):i49-i59. doi: 10.1093/intqhc/mzg085
31. Larcos G, Prgomet M, Georgiou A, et al. A work observation study of nuclear medicine technologists: interruptions, resilience and implications for patient safety. *BMJ Quality & Safety* 2017;26(6):466.
32. Hollnagel E, Woods DD, Leveson N. Resilience Engineering: Concepts and Precepts. Farnham: Ashgate Publishing 2006.
33. Francis R. Report of the Mid Staffordshire NHS Foundation Trust Public Inquiry Executive Summary. London, 2013.
34. Department of Health. An organisation with a memory. London, 2000.
35. National Advisory Group on the Safety of Patients in England. A promise to learn - a comittmnet to act. Improving the safety of patients in England. London, 2013:1-46.
36. Greenhalgh T, Stramer K, Bratan T, et al. The devil's in the detail: final report of the independent evaluation of the Summary Care Record and HealthSpace programmes. London: University College London.
37. National Institute for Health and Care Excellence. Refer-to-Pharmacy and Medicines Optimisation 2017 [Available from: https://www.nice.org.uk/sharedlearning/refer-to-pharmacy-and-medicines-optimisation accessed 25/01/2018 2018.
38. Batalden M, Batalden P, Margolis P, et al. Coproduction of healthcare service. *BMJ quality & safety* 2016;25(7):509-17. doi: 10.1136/bmjqs-2015-004315
39. Krist AH, Woolf SH. A Vision for Patient-Centered Health Information Systems. *JAMA* 2011;305(3):300-01. doi: 10.1001/jama.2010.2011
40. Cousins DH, Gerrett D, Warner B. A review of medication incidents reported to the National Reporting and Learning System in England and Wales over 6 years (2005-2010). *British Journal of Clinical Pharmacology* 2012;74(4):597-604. doi: 10.1111/j.1365-2125.2011.04166.x
41. Merrick Z, Joanne G, Scott R. Interprofessional collaboration: effects of pratice-based interventions on professional pratice and healthcare outcomes. *Cocharane Database of Systematic Reviews In: Cochrane Library* 2014(1):Art. No. CD000072-Art. No. CD72. doi: 10.1002/14651858.CD000072.pub2
42. Rasmussen J. Risk management in a dynamic society: a modelling problem. *Safety Science* 1997;27(2):183-213. doi: https://doi.org/10.1016/S0925-7535(97)00052-0
43. Svedung I, Rasmussen J. Graphic representation of accidentscenarios: mapping system structure and the causation of accidents. *Safety Science* 2002;40(5):397-417. doi: https://doi.org/10.1016/S0925-7535(00)00036-9

For peer review only - http://bmjopen.bmj.com/site/about/guidelines.xhtml

Standards for reporting qualitative research checklist (SRQR)
from O'Brien BC, Harris IB, Beckman TJ, Reed DA, Cook DA. Standards for reporting qualitative research: a synthesis of recommendations. Acad Med. 2014;89(9):1245-1251.

**Title and abstract**			
S1	Title	Concise description of the nature and topic of the study Identifying the study as qualitative or indicating the approach (e.g., ethnography, grounded theory) or data collection methods (e.g., interview, focus group) is recommended	✓ Study identified as mixed methods in the title
S2	Abstract	Summary of key elements of the study using the abstract format of the intended publication; typically includes background, purpose, methods, results, and conclusions	✓ Elements contained within the abstract
**Introduction**			
S3	Problem formulation	Description and significance of the problem/phenomenon studied; review of relevant theory and empirical work; problem statement	✓ Text identifies the risks to heart failure patients through poorly managed medicines
S4	Purpose or research question	Purpose of the study and specific objectives or questions	✓ These are stated at the end of the introduction
**Methods**			
S5	Qualitative approach and research paradigm	Qualitative approach (e.g., ethnography, grounded theory, case study, phenomenology, narrative research) and guiding theory if appropriate; identifying the research paradigm (e.g., postpositivist, constructivist/ interpretivist) is also recommended; rationale	✓Resilience identified as the guiding theory for the analysis
S6	Researcher characteristics and reflexivity	Researchers' characteristics that may influence the research, including personal attributes, qualifications/experience, relationship with participants, assumptions, and/or presuppositions; potential or actual interaction between researchers' characteristics and the research questions, approach, methods, results, and/or transferability	✓ Researchers identified as experienced fieldworkers
S7	Context	Setting/site and salient contextual factors; rationale	✓included on page 4
S8	Sampling strategy	How and why research participants, documents, or events were selected; criteria for deciding when no further sampling was necessary (e.g., sampling saturation);	✓included on page 4
S10	Data collection methods	Types of data collected; details of data collection procedures including (as appropriate) start and stop dates of data collection and analysis, iterative process, triangulation of sources/methods, and modification of procedures in response to evolving study findings; rationale	✓included on pages 4-5
S11	Data collection instruments and technologies	Description of instruments (e.g., interview guides, questionnaires) and devices (e.g., audio recorders) used for data collection; if/how the instrument(s) changed over the course of the study	✓included on page 4-5
S12	Units of study	Number and relevant characteristics of participants, documents, or events included in the study; level of participation (could be reported in results)	✓Included in Table 1
S13	Data processing	Methods for processing data prior to and during analysis, including transcription, data entry, data management and security, verification of data integrity, data coding, and anonymization/deidentification of excerpts	✓ Included in methods

For peer review only

Standards for reporting qualitative research checklist (SRQR)
from O'Brien BC, Harris IB, Beckman TJ, Reed DA, Cook DA. Standards for reporting qualitative research: a synthesis of recommendations. Acad Med. 2014;89(9):1245-1251.

S14	Data analysis	Process by which inferences, themes, etc., were identified and developed, including the researchers involved in data analysis; usually references a specific paradigm or approach; rationale b	✓ Described on page 6
S15	Techniques to enhance trustworthiness	Techniques to enhance trustworthiness and credibility of data analysis (e.g., member checking, audit trail, triangulation); rationale	✓ Described on page 6
**Results / findings**			
S16	Synthesis and interpretation	Main findings (e.g., interpretations, inferences, and themes); might include development of a theory or model, or integration with prior research or theory	✓ included in Results section
S17	Links to empirical data	Evidence (e.g., quotes, field notes, text excerpts, photographs) to substantiate analytic findings	✓ included throughout
**Discussion**			
S18	Integration with prior work, implications, transferability, and contribution(s) to the field	Short summary of main findings; explanation of how findings and conclusions connect to, support, elaborate on, or challenge conclusions of earlier scholarship; discussion of scope of application/ generalizability; identification of unique contribution(s) to scholarship in a discipline or field	✓included in discussion including implications for policy, theory and practice
S19	Limitations	Trustworthiness and limitations of findings	✓Main limitations are documented
**Other**			
S20	Conflicts of interest	Potential sources of influence or perceived influence on study conduct and conclusions; how these were managed	None identified
S21	Funding	Sources of funding and other support; role of funders in data collection, interpretation, and reporting	✓

For peer review only

BMJ Open

Gaps, traps, bridges and props: a mixed-methods study of resilience in the medicines management system for heart failure patients at hospital discharge

Journal:	*BMJ Open*
Manuscript ID	bmjopen-2018-023440.R1
Article Type:	Research
Date Submitted by the Author:	04-Jul-2018
Complete List of Authors:	Fylan, Beth; University of Bradford, School of Pharmacy and Medical Sciences; Bradford Institute for Health Research<br>Marques, Iuri; University of Bradford, School of Pharmacy and Medical Sciences<br>Ismail, Hanif; University of Bradford, School of Pharmacy and Medical Sciences<br>Breen, Liz; University of Bradford, School of Pharmacy and Medical Sciences<br>Gardner, Peter; University of Leeds Institute of Psychological Sciences<br>Armitage, Gerry; Bradford District Care NHS Foundation Trust; University of Bradford, Faculty of Health<br>Blenkinsopp, Alison ; University of Bradford, School of Pharmacy and Medical Sciences
**Primary Subject Heading**:	Cardiovascular medicine
Secondary Subject Heading:	Health services research, Medical management, Pharmacology and therapeutics, Cardiovascular medicine
Keywords:	Resilience, Patient Safety, Heart failure < CARDIOLOGY, Medicines Optimisation, Quality in health care < HEALTH SERVICES ADMINISTRATION & MANAGEMENT

SCHOLARONE™
Manuscripts

Gaps, traps, bridges and props: a mixed-methods study of resilience in the medicines management system for heart failure patients at hospital discharge

Beth Fylan,[1 2] Iuri Marques,[1] Hanif Ismail,[1] Liz Breen, [1] Peter Gardner,[3] Gerry Armitage,[1 4] Alison Blenkinsopp[1]

On behalf of the ISCOMAT Programme Team

[1] University of Bradford, Bradford, UK

[2] Bradford Institute for Health Research, Bradford, UK

[3] University of Leeds, Leeds, UK

[4] Bradford District Care Trust, Bradford, UK

Corresponding author: Dr Beth Fylan, School of Pharmacy and Medical Sciences, Richmond M24, University of Bradford BD7 1DP

Email: B.FylanGwynn@bradford.ac.uk

Tel: 01274 236952

Fax: Not available

Keywords: Patient safety; resilience; cardiology; medicines management

Abstract

Introduction

Poor medicines management places patients at risk, particularly during care transitions. For patients with heart failure (HF), optimal medicines management is crucial to control symptoms and prevent hospital readmission. This study explored the concept of resilience using HF as an example condition to understand how the system compensates for known and unknown weaknesses.

Methods

We explored resilience using a mixed-methods approach in four healthcare economies in the north of England. Data from hospital site observations, healthcare staff and patient interviews, and documentary analysis were collected between June 2016 and March 2017. Data were synthesised and analysed using framework analysis.

Results

Interviews were conducted with 45 healthcare professionals, with 20 patients at three time-points and 189 hours of observation were undertaken. We identified four primary inter-

For peer review only - http://bmjopen.bmj.com/site/about/guidelines.xhtml

For peer review only

related themes concerning organisational resilience. These were named as gaps, traps, bridges and props. Gaps were discontinuities in processes that had the potential to result in poorly optimised medicines. Traps were features of the system that could produce errors or unintended adverse medication events. 'Bridges' were features of the medicines management system that promoted safety and continuity which ensured that, despite varying conditions, care could be delivered successfully. 'Props' were informal, temporary or impromptu actions taken by patients or healthcare staff to avoid potential adverse events.

Conclusion
The numerous opportunities for HF patient safety to be compromised and sub-optimal medicines management during this common care transition are mitigated by system resilience. Cross-organisational bridges and temporary fixes or 'props' put in place by patients and carers, healthcare teams and organisations are critical for safe and optimal care to be delivered in the face of continued system pressures.

For peer review only

Strengths and limitations of the study

- Using four geographical areas and mixed methods, this study explores medicines management for people with heart failure at a time of considerable risk.
- Multiple viewpoints of patients and staff highlighted how the system is resilient in the face of pressure and weaknesses.
- The study presents a framework within which to explore and understand resilience in healthcare systems: that of 'bridges' and 'props' set against a backdrop of 'gaps' and 'traps'.
- The study collected multiple viewpoints but did not include the perspectives of local, regional and national policy makers.
- The patients and staff who agreed to be interviewed may have had particularly positive or negative experiences of the system, although their accounts were augmented and triangulated by first-hand independent observations.

Introduction

The World Health Organisation (WHO) views the safe management of medicines as a global challenge.[1] In the UK, guidance stresses the importance of improving the way medicines are

For peer review only - http://bmjopen.bmj.com/site/about/guidelines.xhtml

managed at care transitions, such as admission to and discharge from hospital.[2] However, there is continued evidence that systems managing medicines are not optimally calibrated, particularly during and after hospital discharge,[3] when the responsibility for patient care shifts across organisations and clinicians.[4]

Medicines management is a system that supports the therapeutic use of medicines by patients, involving multiple healthcare organisations and staff with different clinical specialties and professional roles.[5] There is no shortage of evidence about the points at which healthcare systems fail to provide safe care.[6-9] Patients are not always well prepared to leave hospital and self-manage their ongoing treatment.[10] The effective transfer of sufficient and accurate information between healthcare organisations remains inadequate in many cases[4] and is compounded by boundaries between care providers who may not always have access to the same information about patients' health. It is then unsurprising that discrepancies arise between medicines lists held by different care providers and patients.[3 11 12]

Heart failure (HF) is a chronic progressive condition affecting 900,000 individuals in the UK and is projected to rise significantly with an ageing population.[13] HF is the second most costly condition for the NHS after stroke and is characterised by high rates of readmissions.[14] Heart failure symptoms and disease progression can be controlled through well managed medicines; however, guidelines for their use are not always applied and cardiology medicines can also cause harm, such as kidney injury, if they are not monitored.[15] Hence, the optimal management of medicines when leaving hospital is crucial to enhance quality of life, manage symptoms, prevent deterioration leading to hospital readmission, and reduce mortality.[16]

Current thinking in patient safety has shifted focus from the deconstruction of events leading up to safety incidents (Safety I) to a more positive and proactive view of healthcare systems that identifies and values what goes right as well as pinpointing what goes wrong (Safety II).[17 18] Thus Safety II focuses on preventing error whilst accepting that there is variability in the delivery of healthcare, acknowledging that patients do not always experience harm as a consequence of their care. Instead of reacting when things go wrong, organisations proactively anticipate developments, negative as well as positive. It offers recognition of good performance in the face of uncertainty, valuing flexibility, adaptability, foresight and knowledge of how systems operate.[17 19] This in turn promotes a more dynamic attitude to performance through resilience which is the ability for a system and the individuals therein to

For peer review only - http://bmjopen.bmj.com/site/about/guidelines.xhtml

adjust prior to, during, or following changes or disturbances or in the face of ongoing, sustained pressure.[18 20-22]

This concept of resilience in healthcare has looked at specific points in the patient pathway such as handover of care between staff in one location such as a ward or performing specific roles, [19 23 24] but not at a point of transition between healthcare organisations. Moreover only one previous study has explored how patients can enhance resilience in medicines management at and after hospital discharge through anticipating discrepancies and taking remedial action.[25] No studies to date have explored resilience in medicines management at this care transition from multiple perspectives, including staff and patients and across different healthcare economies.

This study aimed to address this evidence gap by systematically investigating resilience in the medicines management system, using HF as an example condition. More specifically, the study was designed to understand how the system compensates for weaknesses and maximises opportunities in order to deliver safe yet optimal treatment. Its objectives were to explore the system of medicines management in multiple healthcare economies to highlight where resilience exists and identify where improvements to the system can be made to enhance resilience.

Methods

We used a mixed-methods design in four healthcare economies and their local primary care organisations (one comprising two hospitals and three comprising one hospital) in the north of England. Sites were selected to include University teaching hospitals and non-University teaching hospitals in different areas. Data from site observations, staff and patient qualitative interviews, and documentary analysis (discharge letters and organisational and national policies) were collected between June 2016 and March 2017. NHS research ethics committee approval was sought and granted (16/NS/0018).

Patient involvement

A patient researcher was a member of the research team advising on patient recruitment, data collection materials and information and consent forms. The research was overseen by a patient-led steering group including people with heart failure and carers.

Data Collection

Observations

For peer review only - http://bmjopen.bmj.com/site/about/guidelines.xhtml

Following ward-level consent, three experienced health researchers (BF, HI, IM) conducted a total of 189 hours of observations in five cardiology wards and one heart failure clinic. Structured observation schedules developed by the research team informed by previous work[26] were used to record observations. We observed medicines and ward rounds, preparation of information for discharge, patient discharges, as well as any other impromptu medicines-related activities. Unstructured, contemporaneous field notes were taken by the researchers.

Patient recruitment

A quota sample of 4-6 patients in each site was constructed, aiming for at least 16 complete datasets in total in the four areas. Patients were recruited during hospital admission by research nurses in consultation with ward staff. Patients were eligible for the study if they were aged 18 years or older, had capacity to consent, and had been admitted to hospital with a diagnosis of heart failure with reduced ejection fraction (<45%) measured by an echocardiogram within the last five years. Patients also needed to present New York Heart Association (NYHA) Class III symptoms.[27] Research nurses approached eligible patients to introduce the study. Patients were then provided with a participant information leaflet and given the opportunity to ask questions about the study; they were given at least four hours to decide whether or not to take part.

Patient interviews

Patients' experiences with their medicines were explored at three time points: at, or as soon as practicable, after discharge (covering experience from admission to discharge) and then approximately two and six weeks later. The research team developed a semi-structured interview schedule built upon previous work[26] and a review of relevant literature. The schedule comprised questions relating to patients' experiences with their medicines, and prompts and probes were used when relevant. Two researchers conducted the interviews (BF, HI). Interviews lasted up to 60 minutes, took place in patients' homes and were video or audio recorded and transcribed verbatim.

Healthcare staff recruitment

Healthcare professionals with a role in medicines management in primary or secondary care were approached to take part in a semi-structured interview either by research nurses or the study team, using face-to-face communication or by e-mail invitation. A range of healthcare professionals involved in medicines management were selected following ward observations.

For peer review only - http://bmjopen.bmj.com/site/about/guidelines.xhtml

Healthcare staff interviews

An interview schedule was developed by the research team, to explore staff perceptions of safe medicines management. The schedule focused on medicines management processes, staff views on its quality and effectiveness for patients with heart failure in primary and secondary care, and their experiences of medicines management at discharge from secondary to primary care. Staff were given a participant information leaflet describing the study and, if they agreed to take part, an appointment was made to conduct the interview. Interviews lasted up to 60 minutes, were audio-recorded following written consent and transcribed verbatim.

Analysis of key documents

Documents were identified and reviewed including: national guidance on medicines optimisation used in the hospital setting;[2] local policies on medicines management and discharge in the four health economies; case notes and communications such as discharge letters, and any patient information about medicines in use in the four hospitals and available as text. Examples of potential system resilience at care transitions and risks in the system were identified and using a framework that mapped them according to the point in the transition to which they related and to the resilience element (or lack of) they evidenced.[19]

Data analysis

The process of data analysis was iterative and comparative: analysing the first round of interview and observation data as further interviews and observations were undertaken; providing the opportunity to explore emerging themes in greater detail in subsequent fieldwork. The research team met several times both during and following data collection to discuss the data synthesis and analysis method and the emerging themes. Interview data were synthesised through data extraction with the data from observations and documents and the combined data were analysed using the Framework approach,[28] involving detailed familiarisation with the data, identifying themes, interpreting the findings within the context of similar research studies, and considering policy and practice.

Results

A total of 56 interviews with 20 heart failure patients were conducted: 19 at discharge or shortly afterwards (timepoint 1); 19 approximately two weeks after discharge (timepoint 2); and 18 approximately six weeks after discharge (timepoint 3). We were unable to contact one patient from site 1 at time-point 1; at site 2 one patient withdrew from the study after the first interview. One patient from Site 3 was not interviewed at the third time-point due to hospital readmission, and at site 4 one patient was too ill to continue after the second

For peer review only - http://bmjopen.bmj.com/site/about/guidelines.xhtml

interview. Table 1 presents the number of patients interviewed for each site at the different time points. Table 2 outlines the gender and age of interviewed patients.

Table 1: The number of patients interviewed at each time-point by site

Site	Timepoint 1	Timepoint 2	Timepoint 3
**Site 1**	2	3	3
**Site 2**	5	4	4
**Site 3**	6	6	5
**Site 4**	6	6	5
**Total**	**19**	**19**	**18**

Table 2: The gender and age of patients who took part in interviews

Site	Gender	Total	Age range
**Site 1**	Male	2	72-82
	Female	1	53
**Site 2**	Male	5	40-89
	Female	0	0
**Site 3**	Male	5	46-79
	Female	1	69
**Site 4**	Male	4	46-78
	Female	2	69-76

Forty-five interviews (Table 3) were conducted with healthcare professionals: 19 with primary care staff (15 in four GP surgeries, two community pharmacists and two community HF nurses) and 26 with secondary care staff. Table 4 presents the number of healthcare staff interviewed by site.

Table 3: Number of interviews by healthcare staff type

Staff Type	Number of interviews
**GPs**	4
**Practice administrators / data quality managers**	2
**Practice pharmacists**	3
**Practice nurses**	1
**Practice managers**	3

For peer review only - http://bmjopen.bmj.com/site/about/guidelines.xhtml

**Community pharmacists**	2
**Community heart failure nurses**	2
**Clinical care co-ordinators**	1
**Community cardiac nurses**	1
**Cardiologists**	3
**Ward managers**	5
**Staff nurses**	2
**Junior sisters**	1
**Ward pharmacists**	3
**Specialist cardiology pharmacists**	2
**Consultant pharmacists**	1
**Junior doctors**	2
**Specialist heart failure nurses**	3
**Ward administrative staff**	4
**Total**	**45**

For peer review

Table 4: The number of healthcare staff interviewed per site

Site	Primary/community care	Secondary care
**Site 1**	6	7
**Site 2**	4	8
**Site 3**	2	4
**Site 4**	7	7
**Total**	**19**	**26**

We identified four primary inter-related themes concerning organisational resilience and termed these: gaps, traps, bridges and props. Examples representing each theme are shown in Tables 5-8.

‘**Gaps’** were defined as a discontinuity in key processes that form the medicines management system and had the potential to result in poorly optimised medicines. Approaches to preparing discharge information varied across sites, with information sometimes being missed due to a lack of preparation time. Gaps were also evident in the

For peer review only - http://bmjopen.bmj.com/site/about/guidelines.xhtml

information shared with primary care and in the preparation of patients to use their medicines. For the latter, we identified no standardised processes for informing patients about their medicines and, while hospital policies stipulated that patients should be informed, and gave details of the types of information patients should have, there was no guidance on optimal methods for informing patients about their medicines or training, so patients' experiences of receiving medicines were inconsistent and information was deficient for some.

> *Discussions with some nurses during observations revealed that while they were aware of policies in place on what aspects to cover when discussing medicines with patients at discharge, they did not follow them and often rushed these conversations* **[Site 2 - Field notes from ward observations]**

After discharge we found gaps in the continuity of care, for example not all patients had a community pharmacy Medicines Use Review (MUR) because pharmacies did not routinely receive information about the patients' medicines at discharge. Waiting times for specialist staff follow-up varied considerably and were sometimes lengthy, for example waiting times for an appointment with community heart failure specialist nurses who would manage medicines titration was sometimes as long as three months after discharge.

We defined '**traps'** as features of the way the medicines management system was designed or managed that might produce medication errors defined as a 'failure in the treatment process that leads to, or has the potential to lead to, harm to the patient'.[29 30] or unintended adverse medication events. These were evident in the co-ordination of discharges, for example the pressure on ward staff to expedite discharges as quickly as possible appeared to impact on the effective preparation of discharge information and on educating patients about their discharge medicines.

> *If you're busy, you'll write less and I think that's just what happens on the job. If I know I've got time, I'll make sure I input as much detail as possible, but if you're busy you just don't have the time to do that, so you'll just really do short summaries and just include the bare essentials.* **(Site 1 FY1 doctor)**

Staff preparing discharge information were often interrupted, could not always locate patients' notes and none reported receiving training about safe practices with medicines at discharge to primary care. We also found error traps after discharge, such as a lack of time and resources in GP surgeries to process discharge information. Finally, there was evidence that patients' lack of knowledge about the purpose of their medicines could potentially cause

For peer review only - http://bmjopen.bmj.com/site/about/guidelines.xhtml

confusion particularly when the changes made in hospital led patients to have different supplies or multiple multi-compartment 'compliance aid' tablet boxes. Like hospital staff, none of the primary care staff had received formal training about safe practices with medicines at discharge to primary care.

'**Bridges'** were identified as formalised features of the medicines management system that had been made permanent and promoted the safety and continuity of medicines management. They ensured that, despite varying conditions, care could be delivered successfully to heart failure patients.

> *When preparing the "To Take Home" medicines at discharge, ward staff wait for the pharmacist to come to the ward to check the patients' medicines lists and ensure these are accurate and any errors can be rectified.* **[Site 2 - Field notes from ward observations]**

Bridges also included methods of communicating with primary care about treatment, for example, when hospitals sent an electronic copy of the patient's discharge summary to their general practitioner (GP). In this case, summaries were put together by multidisciplinary teams including junior doctors, nurses and pharmacists who would check and add information about medicines that would be useful to the primary care team. After discharge, two participating hospital trusts ran pharmacist-led titration clinics to ensure that medicine doses were optimised. Titration clinics also meant patients would be seen more quickly than if they had to see a consultant cardiologist. One cardiology pharmacist explained that the titration clinic ensured patients' medicines were adjusted as and when appropriate, in light of some GPs not feeling confident about changing them.

GP practices differed in how they processed discharge information. In one practice, administrative staff would review the discharge summary and forward actions to practice staff if medicines information needed to be changed. In another practice, this task was the responsibility of the GP, who would forward actions to practice staff and book any tests needed as a consequence of any changes in medicines occurring during hospital stay (i.e. blood tests). One GP reported that his practice had re-engineered their processing of discharge summaries to include a multidisciplinary team comprising administrative staff, a practice-based pharmacist (whose post was created in response to recognised safety risks) and GPs, with the pharmacist taking responsibility for coordinating the process.

> *"And so [processing discharge summaries] it was in-between surgeries, it was at the end of the day, so it was being fitted in rather than having allocated time, so naturally when it's being fitted in the process is a bit more rushed, you're more under pressure,*

For peer review only - http://bmjopen.bmj.com/site/about/guidelines.xhtml

maybe your concentration levels aren't there, so mistakes can be easily made. So as a practice we made the decision that just in terms of a workload thing and also patient safety and efficiency it would be worth investing in sort of pharmacy services." **[GP, Site 1]**

Practice pharmacists perceived that their specialist knowledge improved as a consequence of being involved in the discharge process, while further expediting the safe management of medicines for patients after discharge. Some practice staff described having targets in place linked to time taken to process discharge summaries, with some practices prioritising processing driven by the risk of readmission. One data quality manager explained that they tried to process discharges within 24 hours of receiving information from the hospital, including reconciling medicines, but also explained that they had a maximum of a week to complete it.

"So we have a week turnaround in order to get any meds reconciliation done. We generally get our electronic discharge normally within 24 hours of the patient being discharged, that would be scanned through the system that will then go to the doctor, the doctor will then forward it to me generally for coding and also to our practice pharmacist." **[Data quality manager, Site 2]**

'**Props'** were informal**,** temporary or impromptu actions taken by patients or healthcare staff to avoid potential adverse events, such as medication errors. Props were sometimes developed in response to risks in the working environment, such as interruptions during medicines rounds.

During medicines rounds, nurses are frequently interrupted whilst sorting patients' medicines. One nurse observed also uses the strategy of signing the drug chart soon as one medicine is sorted into the plastic cup before moving to the next medicine. If there are interruptions, the nurse will know which medicines have already been sorted by looking at the drug chart. The nurse says it is a brilliant strategy to ensure accuracy and safety and cope with inevitable distractions and interruptions **[Site 1, Field notes from ward observations]**

Hospital staff told us that they suspected recommendations made by the hospital (for example the up-titration of doses which is critical in HF) may not be acted on in primary care. Hence, staff created solutions to prevent a break in the ongoing treatment, giving patients an extra copy of their discharge letter to take to the GP. Some staff members described being cognisant of how discharge information can be difficult for patients to understand and would take extra time to explain the discharge summary and any abbreviations contained within it. One staff nurse at Site 1 described having to make protected time to hold these discussions with patients, drawing curtains around the patients' beds to prevent any disruption. Some patients reported being discharged with an insufficient amount of medicines, leading them to

For peer review only - http://bmjopen.bmj.com/site/about/guidelines.xhtml

seek community pharmacists help to provide them with emergency supplies until they could see a GP. Some patients also proactively provided the necessary links between community pharmacy, general practitioners and the hospital after discharge. For example, one patient called the community pharmacy to ask what information, if any, they had been provided with about his medicines. Another patient provided their GP practice with information about dose changes.

> *So when I'd run out, I rang my GP and they were blissfully unaware of any changes to the amount, the receptionist had to take it down. She says "well what was you on?" and I said "well I was on one tablet a day and then they took me down to half, then they put me to one tablet a day again and now I'm on two tablets a day" "Two tablets?", this is the receptionist's questions. I says "yeah, two tablets."* **[Patient 05, Site 4, interview 2]**

Finally, community pharmacists stepped in to organise supplies for patients when something had gone wrong and the patient was unable to get the correct medicines.

For peer review only

Table 5: Gaps at and after hospital discharge

	At discharge	After discharge
**Gaps**	Discussions about medicines at discharge can be rushed, due to time pressures and workload.	Community pharmacy is not integrated into communication about discharge medicines
	No standard process or guidance on how to hold discussions with patients about medicines	Patients are not routinely referred to community pharmacy for follow-up support
	Limited or no formal training about care transitions, preparing discharge summaries or patients to use medicines for all staff	Limitations to the extent of shared IT systems between primary and secondary care and between surgeries and pharmacies
	Processes for preparing patients to go home with medicines are linear but not streamlined, for example multiple staff members need to input which causes delays	Not all surgeries have a practice pharmacist to reconcile medicines
	Discharge summary information is technical and uses jargon and abbreviations which are difficult for patients to understand	Long waiting times to access community heart failure nurse services (up to 12 weeks)
	Inconsistency in level of detail in information written on discharge summary due to workload and healthcare staff knowledge of the patient	Some patients perceive limitations in post-hospital follow-up care, including difficulty in accessing services in primary care
	Varying information offered to patients about follow-up appointments	Patients are not fully aware of the roles and skills of primary care staff, particularly community pharmacists
	Limited awareness among staff about policies in place for medicines management	Some patients unable to devise effective strategies to self-manage medicines at home
	Effectiveness of discharge are not critically appraised due to lack of feedback (unless the patient is	

For peer review only

For peer review only - http://bmjopen.bmj.com/site/about/guidelines.xhtml

	readmitted or primary care staff make queries)	

Table 6: Traps at and after hospital discharge

	At Discharge	After discharge
**Traps**	Patient knowledge of medicines when they are discharged is limited	Community pharmacy does not routinely receive copies of patients' discharge summaries so cannot correct or query new GP prescriptions
	There is pressure on ward staff to discharge patients and free-up beds	Patients have an on-going lack of knowledge of their medicines once home
	Variation in ward staffing levels and varying numbers of discharges to perform each day	No formal training for surgery staff to process discharge information
	Use of several different IT systems in producing information for discharge	Lack of time and resources in surgery to process discharge information
	Staff preparing patients for discharge and information about discharge medicines are interrupted	Systems allow old prescriptions to be issued when medicines have changed
	Preparing information for discharge routinely left to junior members of staff who may not be familiar with the patient	Dosages are monitored and changed by staff in different organisations
	Conversations about medicines with patients at discharge can be left to the last minute	Trust in healthcare professionals may lead to a lack of critical appraisal of one's condition and medicines
	Patients transferred to discharge lounges to await medicines face an extra transfer of care	Changes in medicines lead to patients having conflicting medicines and multi compartment compliance aid boxes at home
		Varying levels of communication across care organisations results in extra burden to patient who has to fill in the gaps
		Varying information about medicines changes provided to primary care may lead to healthcare staff having to make decisions based on assumptions
		Healthcare professions may not accept treatment recommendation by other healthcare professionals (e.g. GP not accepting recommendations made by HFSN)

For peer review only - http://bmjopen.bmj.com/site/about/guidelines.xhtml

Table 7: Bridges at and after discharge

	At discharge	After discharge
**Bridges**	Hospitals have established methods of communicating about patients' treatment with primary care	Some trusts provide outpatient clinics where patients can receive IV fluids, thus avoiding them to need to be admitted to receive these medicines or speeding up discharges
	Preparing discharge summaries and To Take Out (TTO) lists is a multidisciplinary task involving nurses and pharmacists	GP practices have systems for acting upon discharge information once it is received, although processes and times to process this information vary
	Ward pharmacists can expedite well managed discharge through proactively creating TTO lists	Some practices have targets in place linked to time to process discharge information (e.g. 24h from receiving this information)
	One trust routinely referred patients to community pharmacy for follow-up support with their medicines	One practice pharmacist reengineered the process for action on discharge information
	All hospitals had policies for informing patients about their medicines	Some practices use practice pharmacists to improve an expedite the processing of discharge information
	Heart failure nursing staff attempted to see patients before their discharge to talk about their medicines to avoid having these conversations rushed at discharge	Community pharmacy is sometimes able to perform post-discharge Medicines Use Reviews for Patients
	In two trusts, ward-based pharmacists would speak to patients about their medicines before discharge	Two hospital trusts run pharmacist-led titration clinics to manage patients' medicines, meaning that patients can be seen and followed-up quickly
	Patients received written information about their medicines, with one trust providing an easy-to-understand medicines chart occasionally annotated by staff	Some practices have ambulatory services
	Patients are referred to specialist heart failure teams for follow-up	Heart failure specialist nurses offer support services including medicines optimisation
		Some GP practices have systems to identify discharged patients with high risk of being readmitted so they can take preventative action

For peer review only

Table 8: Props at and after hospital discharge

	At discharge	After discharge
**Props**	Some staff create their own checklists to follow discharge processes, such as using the discharge summary to tick off medicines	Patients create their own lists of medicines, going online to seek more information
	Staff occasionally give patients two copies of the discharge summary so that patients can give one to their GPs in case they do not receive it electronically	Community pharmacists who have received a copy of the discharge summary use them to check against repeat prescriptions before dispensing
	Staff make ad-hoc queries to establish reasons for medicines changes which are unclear and undocumented so that they can be clear on the discharge summary	Patients check medicines prescribed by their GPs against their discharge summary and/or take a copy when go see the GP or update them verbally
	Staff will delay discharge to wait for relatives to arrive so that they can include them in conversations about medicines	GP identifying potentially problematic changes in medicines occurring in hospital due to their enhanced knowledge of the patient
	Ward pharmacists give advice to patients if they are concerned about patients getting confused, for example, advising them to return their old medicines to the pharmacy for disposal and only take the new ones	GPs try to fill in patients' knowledge gaps about their medicines after discharge
	Patients write additional information on the medicines' boxes or ask staff to write it so that they can better manage their medicines at home, for example time to take medicines	Community pharmacy provides emergency supply of medicines when patients are discharged from hospital without sufficient medicines
	Patients are sometimes cognisant of how difficult it is for patients to understand their medicines and information provided at discharge, so they take extra time to hold these conversations	Patients are given telephone numbers for heart failure nurses to contact them after discharge because waiting times to be seen by them are long
	Staff draw curtains around the patients' beds when talking to them to ensure privacy and prevent interruptions	Heart failure nurses can identify where patients make mistakes taking their medicines, for example, continuing to take discontinued medicines
	Nurses resist instructions to send patients to discharge lounges as they feel the staff will not have specialist knowledge, and provide enhanced instructions to discharge lounge if overruled	Heart failure nurses use the patients as a conduit for information to be exchanged between them and other healthcare professionals

For peer review only - http://bmjopen.bmj.com/site/about/guidelines.xhtml

	Junior doctors query with pharmacist on ward if they need additional information about medicines	Patients develop individual strategies and routines to adhere to medicines at home, for example alarms, writing additional information in the discharge summary, storage systems, affixing discharge summaries on the fridge, etc
		Some patients take all their medicines to community pharmacy after discharge, seeking information on which medicines they should continue to take and which should be discarded

For peer review only

For peer review only - http://bmjopen.bmj.com/site/about/guidelines.xhtml

Discussion

Notwithstanding a considerable body of research that has illuminated the sometimes alarming levels of preventable harm in healthcare systems and how this could be reduced,[31 32] this study suggests that there are opportunities to enhance the system that manages medicines across multiple organisations. The study also provides a positive perspective on the strategies developed and actions taken by healthcare organisations, their staff, and patients to provide care successfully in the face of continued pressure and gaps that appear between and between organisations in this complex system.[33] The multiple perspectives of patients and multidisciplinary staff, independent observation of practice and documentary analysis collected through mixed-methods allowed the possibility of triangulating data from multiple sources to offer a thorough description of a complex system. In doing so we also present a framework within which to explore and understand resilience in healthcare systems: that of 'bridges' and 'props' set against a backdrop of 'gaps' and 'traps'. Moreover, this study explores a whole healthcare system inclusive of its transitions, to reveal the context of that system. In contrast, previous studies although adding to an understanding of system resilience, have examined these problems from solely a health professional perspective[23] or a patient perspective.[25]

Bridges and props either provided permanent system adaptations to potential gaps in care, or temporary fixes, usually implemented by individuals or small teams. Sometimes the props were put in place despite organisational pressure, for example to discharge patients and free beds. We also draw out the dissonance between what healthcare professionals believe should happen and the reality of contemporary practice. This was clear from the differences between the recommendations for hospital discharge from national guidance and local policy, where, for example, patients must be fully informed about their medicines and any changes, and the overall discharge process – which in the settings observed, may lack depth and the necessary detail, or appear rushed. This was sometimes due to different local conditions, such as the number of discharges that needed to be completed in a day, but also to local policies that lacked sufficient detail and were not supported by staff training. Healthcare systems are complex and non-linear and the Safety II paradigm asserts that success and failures are products of the same variable system performance and that linear models of events such as medication errors cannot reflect the complexity of modern healthcare systems.[34] An enhanced view of the system using a Safety II lens allows healthcare organisations and policy-makers to understand and close the gap between work as imagined versus work as done.[35] This view also provides a better understanding of how policies and guidelines are actually interpreted and whether they are implemented in

For peer review only - http://bmjopen.bmj.com/site/about/guidelines.xhtml

healthcare organisations by staff who adjust their performance to deliver care in a complex system.

Resilient systems are able to learn from their clinical experience (both positive and negative, positive and negative), adapt to it and respond to provide successful outcomes.[36 37] It was evident that staff were able to anticipate system vulnerabilities, for example in the transfer of discharge information, and take compensatory adaptive action in the form of 'props'. As found by a previous study, patients also took remedial action, such as providing missing information about medicines changes to staff.[25] Resilient systems can monitor, learn and anticipate opportunities to improve. A better understanding and acceptance of the error traps in the system present healthcare organisations with the opportunity to learn about how the system operates, particularly when it is under pressure and presents a basis to improve. A better knowledge of gaps allows staff to anticipate where problems may occur and take action to avoid them. Props in the system are indicators of how flexible staff and teams are and healthcare systems can learn from the temporary fixes put in place and knowing where bridges have successfully joined up care can help systems learn and be better placed to innovate elsewhere. There are opportunities to learn from the 'ordinary performance adjustments' that staff undertake to better understand how to keep patients safe, [37] thereby formalising system props into bridges.

Implications for policy and practice

Successive UK government-commissioned reports have highlighted how care systems have failed and how the actions – or inactions of those who lead or contribute to the system – have sometimes led to poor care and patient harm.[38-40] Policymakers should recognise the attempts made routinely by healthcare professionals and teams to learn from their clinical experience and apply this learning to increase system resilience by delivering safer care for patients despite disruptive conditions, such as disconnected communication systems, varying staffing levels and the under-provision of formal training, for example in discharge and care transfers. Our study has shown that improvements to both the efficiency and safety of care could be gained through connecting the discrete IT systems that operate within and between organisations. Additionally, community pharmacists often remain isolated from the patient pathway and are not routinely included in the communication between secondary care and primary care practice, creating additional risk for heart failure patients who must obtain new supplies of critical medicines often within one or two weeks of being discharged.[4] Implementing systems that enable community pharmacists to know about medicines changes made during hospital admissions and thus to reconcile subsequent GP prescriptions would improve safety of medicines management, especially for heart failure

For peer review only - http://bmjopen.bmj.com/site/about/guidelines.xhtml

patients whose medicines are very commonly changed following a period of acute care. Local electronic systems do exist in a small number of areas to ensure that the dispensers of post-discharge medicines are fully informed about the medicines hospital clinicians intended patients should take so that they can reconcile those medicines and ensure accurate ongoing supplies.[41] Policy makers also have a duty to help disseminate and promote implementation of these local innovations – such as the transfer of discharge medicines information to all agents in the medicines management system – which minimise inherent risk.

Patients, if they so desire, should also be provided with the opportunity to gain in-depth knowledge of their medicines before leaving hospital (or afterwards if they prefer), in order to enhance their ability to self-manage and monitor their condition; such knowledge might also increase patients' vigilance, their capacity for error detection, and therefore to ask for prompt support if medication problems arise. Materials to support patients should be developed using co-design methods to maximise their acceptability and usability with both patients and healthcare staff.[42] Policy makers may also consider allowing patients to write to and share a personal health record to keep track of and flag problems they may have with their medicines, and share these with their healthcare teams and report them to their care providers.[43] This would in some measure help address the underreporting of medication errors, particularly in primary care.[44]

We found that staff received little formal training in co-ordinating medicines management, including in completing discharge summaries, and there was little evidence of inter-professional or cross-pathway training. Such training may foster a care environment where clinical and administrative staff have a better appreciation of the impact of the care they provide on different parts of the system, and on different colleagues. For example, how inadequate information on a discharge summary can cause difficulties for primary care staff attempting to reconcile medicines. Additionally, in primary care, understanding that the processing of discharge information can impact on patients and community pharmacists who must take action to ensure the correct medicines are supplied. Inter-professional education has been found to yield positive outcomes in healthcare and may be especially helpful here, although more evidence for its effectiveness has been called for.[45]

Implications for future research

The Safety I paradigm produced valuable ways of unearthing and visualising risks within systems and explaining causation when accidents occur.[32] In healthcare systems, Safety II can add substantially by focusing on how safe care is delivered in the face of disruption and

For peer review only - http://bmjopen.bmj.com/site/about/guidelines.xhtml

pressure by way of bridges, props or both, from individual, micro (e.g. healthcare teams) and macro (e.g. organisational) perspectives. Investigating further how this happens for different health conditions using tailored methodologies will allow a better understanding of safe, resilient care, and afford commissioners a view of how changes to services may impact on a complex system.[46]

.

Limitations

We observed practice in four NHS Trusts and interviewed a wide-range of healthcare staff across the pathway and patients, alongside reviewing key documents, we did not include the perspectives of local, regional and national policy makers, which may have enhanced the understanding of how systems are designed and the gaps between design and delivery. Nevertheless we were able to collect a large amount of data to compare policy practice which enhanced reliability and validity. The patients and staff who agreed to be interviewed may have had particularly positive or negative experiences of the system, although their accounts were triangulated by first-hand independent observations. Finally, the study was conducted in four NHS healthcare economies, at a time of heightened focus on the quality of healthcare, and reports of unprecedented financial constraints, which may have impacted on people's perspectives of care received and delivered, and on the nature of the care observed.

Conclusion

There are numerous opportunities for patient safety to be compromised and medicines to be sub-optimally managed during this care transition. However, there are also cross-organisational bridges and temporary fixes in the form of props, put in place by individuals, including patients an carers, and teams to maximise the opportunity for safe and optimal care to be delivered.. Investigating gaps and traps in the healthcare system and identifying existing compensatory props and bridges allow the illustration of areas where healthcare can be improved and fragmented communication minimised during care transitions.

Acknowledgements

We would like to thank the patients and NHS staff who took part in this research. We also with to thank other members of the ISCOMAT programme management team.

Funding statement

For peer review only - http://bmjopen.bmj.com/site/about/guidelines.xhtml

This study was funded by the National Institute for Health Research Programme Grants for Applied Research 'Improving the safety and continuity of medicines management at care transitions' (ISCOMAT) RP-PG-0514-20009. The views expressed are those of the authors and not necessarily those of the NHS, the NIHR or the Department of Health and Social Care.

Data sharing statement

The transcripts of interviews are not available at the Dryad repository

Contributors

BF drafted the protocol, developed data collection tools, conducted fieldwork, analysed the data and drafted the manuscript. IM conducted fieldwork, analysed the data and drafted the manuscript. HI conducted fieldwork, analysed the data and commented on the manuscript. LB advised on data analysis. PG drafted the protocol, directed the study, advised on data analysis and commented on the manuscript. GA designed the study, drafted the protocol, directed data analysis and drafted the manuscript. AB designed the study, drafted the protocol and edited the manuscript.

Competing interests

None declared.

References

1. The World Health Organization. Medication Without Harm: WHO Global Patient Safety Challenge: The World Health Organization, 2017.
2. National Institute for Health and Care Excellence. Medicines optimisation: the safe and effective use of medicines to enable the best possible outcomes (NICE guideline 5), 2015.
3. Coleman E, Smith JD, Raha D, et al. Posthospital Medication Discrepancies. Prevalence and Contributing Factors. *Archives of internal medicine* 2005;165:1842-47.
4. Urban R, Paloumpi E, Rana N, et al. Communicating medication changes to community pharmacy post-discharge: the good, the bad, and the improvements. *International journal of clinical pharmacy* 2013;35(5):813-20. doi: 10.1007/s11096-013-9813-9
5. Phipps DL, Morris RL, Blakeman T, et al. What is involved in medicines management across care boundaries? A qualitative study of healthcare practitioners' experiences in the case of acute kidney injury. *BMJ Open* 2017;7(1):e011765. doi: 10.1136/bmjopen-2016-011765
6. Leape LL, Brennan TA, Laird N, et al. The nature of adverse events in hospitalized patients. Results of the Harvard Medical Practice Study II. *The New England journal of medicine* 1991;324(6):377-84. doi: 10.1056/NEJM199102073240605
7. Brennan TA, Leape LL, Laird NANM, et al. Special Articles Incidence of Adverse Events and Negligence in Hospitalized Patients Results of The Harvard Medical Practice Study I. *The new England Journal of Medicine* 1991(Appendix I):370-76.
8. Landrigan CP, Parry GJ, Bones CB, et al. Temporal trends in rates of patient harm resulting from medical care. *The New England journal of medicine* 2010;363(22):2124-34. doi: 10.1056/NEJMsa1004404
9. Waring J, Bishop S, Marshall F. A qualitative study of professional and carer perceptions of the threats to safe hospital discharge for stroke and hip fracture patients in the English National Health Service. *BMC Health Serv Res* 2016;16:297. doi: 10.1186/s12913-016-1568-2

For peer review only - http://bmjopen.bmj.com/site/about/guidelines.xhtml

10. Knight D, Thompson D, Mathie E, et al. 'Seamless care? Just a list would have helped!' Older people and their carer's experiences of support with medication on discharge home from hospital. *Health Expectations* 2013;16(3):277-91. doi: 10.1111/j.1369-7625.2011.00714.x
11. Duggan C, Feldman R, Hough J, et al. Reducing Adverse Prescribing Discrepancies Following Hospital Discharge. *International Journal of Pharmacy Practice* 1998;6(2):77-82. doi: 10.1111/j.2042-7174.1998.tb00920.x
12. Morcos S. Where are the weakest links?: A descriptive study of discrepancies in prescribing between primary and secondary sectors of mental health service provision. *Psychiatric Bulletin* 2002;26(10):371-74. doi: 10.1192/pb.26.10.371
13. Taylor CJ, Ryan R, Nichols L, et al. Survival following a diagnosis of heart failure in primary care. *Fam Pract* 2017;34(2):161-68. doi: 10.1093/fampra/cmw145
14. Stewart S, Jenkins A, Buchan S, et al. The current cost of heart failure to the National Health Service in the UK. *European Journal of Heart Failure* 2002;4:361-71. doi: 10.1016/S1388-9842(01)00198-2
15. Michaels AD, Spinler SA, Leeper B, et al. Medication Errors in Acute Cardiovascular and Stroke Patients: A Scientific Statement From the American Heart Association. *Circulation* 2010;121(14):1664-82.
16. Koshman SL, Charrois TL, Simpson SH, et al. Pharmacist Care of Patients With Heart Failure. *Archives of Internal Medicine*;168(7):687-94.
17. Braithwaite J, Wears RL, Hollnagel E. Resilient health care: turning patient safety on its head. *Int J Qual Health Care* 2015;27(5):418-20. doi: 10.1093/intqhc/mzv063
18. Hollnagel E. Making health care resilient: From Safety-I to Safety II. In: Hollnagel E, Braithwaite J, Wears RL, eds. Farnham: Ashgate Publishing Limited 2013.
19. Jeffcott SA, Ibrahim JE, Cameron PA. Resilience in healthcare and clinical handover. *Quality & safety in health care* 2009;18(4):256-60. doi: 10.1136/qshc.2008.030163
20. Hollnagel E. Safety-I and safety-II: the past and future of safety management. Farnham: Ashgate Publishing Limited 2013.
21. Hollnagel E. Resilience Engineering in Practice - A guidebook. Farnham: Ashgate Publishing Limited 2010.
22. Barasa EW, Cloete K, Gilson L. From bouncing back, to nurturing emergence: reframing the concept of resilience in health systems strengthening. *Health Policy Plan* 2017;32(suppl_3):iii91-iii94. doi: 10.1093/heapol/czx118
23. Larcos G, Prgomet M, Georgiou A, et al. A work observation study of nuclear medicine technologists: interruptions, resilience and implications for patient safety. *BMJ Quality & Safety* 2017;26(6):466.
24. Furniss D, Barber N, Lyons I, et al. Unintentional non-adherence: can a spoon full of resilience help the medicine go down? *BMJ quality & safety* 2014;23(2):95-8. doi: 10.1136/bmjqs-2013-002276
25. Fylan B, Armitage G, Naylor D, et al. A qualitative study of patient involvement in medicines management after hospital discharge: an under-recognised source of systems resilience. *BMJ Qual Saf* 2017
26. Fylan Gwynn E. Medicines management after hospital discharge: patients' personal and professional networks. University of Bradford, 2015.
27. Zile MR. New Concepts in Diastolic Dysfunction and Diastolic Heart Failure: Part I: Diagnosis, Prognosis, and Measurements of Diastolic Function. *Circulation* 2002;105(11):1387-93. doi: 10.1161/hc1102.105289
28. Ritchie J, Lewis J. Qualitative research practice: a guide for social science students and researchers. London: Sage 2003.
29. Aronson JK. Medication errors: definitions and classification. *British journal of clinical pharmacology* 2009;67(6):599-604. doi: 10.1111/j.1365-2125.2009.03415.x
30. Ferner RE, Aronson JK. Clarification of Terminology in Medication Errors: Definitions and Classification. *Drug Safety* 2006;29(11):1011-22.
31. Classen DC, Pestonik MS, Evans RS, et al. Adverse Drug Events in Hospitalized Patients Excess Length of Stay, Extra Costs, and Attributable Mortality. *JAMA* 1997;277(4):301-06.
32. Runciman WB, Roughead EE, Semple SJ, et al. Adverse drug events and medication errors in Australia. *International Journal for Quality in Health Care* 2003;15(Supplement 1):i49-i59. doi: 10.1093/intqhc/mzg085
33. Cook RI, Render M, Woods DD. Gaps in the continuity of care and progress on patient safety. *BMJ (Clinical research ed)* 2000;320(7237):791-94. doi: 10.1136/bmj.320.7237.791

For peer review only - http://bmjopen.bmj.com/site/about/guidelines.xhtml

34. Hollnagel E. Is safety a subject for science? *Safety Science* 2014;67:21-24. doi: 10.1016/j.ssci.2013.07.025
35. Hollnagel E. Why is work-as-imagined different from work-as-done? In: Wears RL, Hollnagel E, Braithwaite J, eds. Resilient Health Care, Volume 2: The Resilience of Everyday Clinical Work. Farham: Ashgate Publishing Limited 2015.
36. Hollnagel E, Woods DD, Leveson N. Resilience Engineering: Concepts and Precepts. Farnham: Ashgate Publishing 2006.
37. Sujan MA, Huang H, Braithwaite J. Learning from incidents in health care: Critique from a Safety-II perspective. *Safety Science* 2017;99:115-21. doi: 10.1016/j.ssci.2016.08.005
38. Francis R. Report of the Mid Staffordshire NHS Foundation Trust Public Inquiry Executive Summary. London, 2013.
39. Department of Health. An organisation with a memory. London, 2000.
40. National Advisory Group on the Safety of Patients in E. A promise to learn - a comittmnet to act. Improving the safety of patients in England. London, 2013:1-46.
41. National Institute for Health and Care Excellence. Refer-to-Pharmacy and Medicines Optimisation 2017 [Available from: https://www.nice.org.uk/sharedlearning/refer-to-pharmacy-and-medicines-optimisation accessed 25/01/2018 2018.
42. Batalden M, Batalden P, Margolis P, et al. Coproduction of healthcare service. *BMJ quality & safety* 2016;25(7):509-17. doi: 10.1136/bmjqs-2015-004315
43. Krist AH, Woolf SH. A Vision for Patient-Centered Health Information Systems. *JAMA* 2011;305(3):300-01. doi: 10.1001/jama.2010.2011
44. Cousins DH, Gerrett D, Warner B. A review of medication incidents reported to the National Reporting and Learning System in England and Wales over 6 years (2005-2010). *British Journal of Clinical Pharmacology* 2012;74(4):597-604. doi: 10.1111/j.1365-2125.2011.04166.x
45. Merrick Z, Joanne G, Scott R. Interprofessional collaboration: effects of pratice-based interventions on professional pratice and healthcare outcomes. *Cocharane Database of Systematic Reviews In: Cochrane Library* 2014(1):Art. No. CD000072-Art. No. CD72. doi: 10.1002/14651858.CD000072.pub2
46. Hollnagel E. FRAM: The Functional Resonance Analysis Method. London: CRC Press 2012.

For peer review only

For peer review only - http://bmjopen.bmj.com/site/about/guidelines.xhtml

Standards for reporting qualitative research checklist (SRQR)
from O'Brien BC, Harris IB, Beckman TJ, Reed DA, Cook DA. Standards for reporting qualitative research: a synthesis of recommendations. Acad Med. 2014;89(9):1245-1251.

**Title and abstract**			
S1	Title	Concise description of the nature and topic of the study Identifying the study as qualitative or indicating the approach (e.g., ethnography, grounded theory) or data collection methods (e.g., interview, focus group) is recommended	✓ Study identified as mixed methods in the title
S2	Abstract	Summary of key elements of the study using the abstract format of the intended publication; typically includes background, purpose, methods, results, and conclusions	✓ Elements contained within the abstract
**Introduction**			
S3	Problem formulation	Description and significance of the problem/phenomenon studied; review of relevant theory and empirical work; problem statement	✓ Text identifies the risks to heart failure patients through poorly managed medicines
S4	Purpose or research question	Purpose of the study and specific objectives or questions	✓ These are stated at the end of the introduction
**Methods**			
S5	Qualitative approach and research paradigm	Qualitative approach (e.g., ethnography, grounded theory, case study, phenomenology, narrative research) and guiding theory if appropriate; identifying the research paradigm (e.g., postpositivist, constructivist/ interpretivist) is also recommended; rationale	✓Resilience identified as the guiding theory for the analysis
S6	Researcher characteristics and reflexivity	Researchers' characteristics that may influence the research, including personal attributes, qualifications/experience, relationship with participants, assumptions, and/or presuppositions; potential or actual interaction between researchers' characteristics and the research questions, approach, methods, results, and/or transferability	✓ Researchers identified as experienced fieldworkers
S7	Context	Setting/site and salient contextual factors; rationale	✓included on page 4
S8	Sampling strategy	How and why research participants, documents, or events were selected; criteria for deciding when no further sampling was necessary (e.g., sampling saturation);	✓included on page 4
S10	Data collection methods	Types of data collected; details of data collection procedures including (as appropriate) start and stop dates of data collection and analysis, iterative process, triangulation of sources/methods, and modification of procedures in response to evolving study findings; rationale	✓included on pages 4-5
S11	Data collection instruments and technologies	Description of instruments (e.g., interview guides, questionnaires) and devices (e.g., audio recorders) used for data collection; if/how the instrument(s) changed over the course of the study	✓included on page 4-5
S12	Units of study	Number and relevant characteristics of participants, documents, or events included in the study; level of participation (could be reported in results)	✓Included in Table 1
S13	Data processing	Methods for processing data prior to and during analysis, including transcription, data entry, data management and security, verification of data integrity, data coding, and anonymization/deidentification of excerpts	✓ Included in methods

For peer review only

Standards for reporting qualitative research checklist (SRQR)
from O'Brien BC, Harris IB, Beckman TJ, Reed DA, Cook DA. Standards for reporting qualitative research: a synthesis of recommendations. Acad Med. 2014;89(9):1245-1251.

S14	Data analysis	Process by which inferences, themes, etc., were identified and developed, including the researchers involved in data analysis; usually references a specific paradigm or approach; rationale b	✓ Described on page 6
S15	Techniques to enhance trustworthiness	Techniques to enhance trustworthiness and credibility of data analysis (e.g., member checking, audit trail, triangulation); rationale	✓ Described on page 6
**Results / findings**			
S16	Synthesis and interpretation	Main findings (e.g., interpretations, inferences, and themes); might include development of a theory or model, or integration with prior research or theory	✓ included in Results section
S17	Links to empirical data	Evidence (e.g., quotes, field notes, text excerpts, photographs) to substantiate analytic findings	✓ included throughout
**Discussion**			
S18	Integration with prior work, implications, transferability, and contribution(s) to the field	Short summary of main findings; explanation of how findings and conclusions connect to, support, elaborate on, or challenge conclusions of earlier scholarship; discussion of scope of application/ generalizability; identification of unique contribution(s) to scholarship in a discipline or field	✓included in discussion including implications for policy, theory and practice
S19	Limitations	Trustworthiness and limitations of findings	✓Main limitations are documented
**Other**			
S20	Conflicts of interest	Potential sources of influence or perceived influence on study conduct and conclusions; how these were managed	None identified
S21	Funding	Sources of funding and other support; role of funders in data collection, interpretation, and reporting	✓

For peer review only
